# Perceived parental permissiveness toward gambling and risky behaviors in adolescents

**DOI:** 10.1556/JBA.3.2014.012

**Published:** 2014-04-05

**Authors:** ROBERT F. LEEMAN, JULIE A. PATOCK-PECKHAM, RANI A. HOFF, SUCHITRA KRISHNAN-SARIN, MARVIN A. STEINBERG, LOREEN J. RUGLE, MARC N. POTENZA

**Affiliations:** ^1^Department of Psychiatry, Yale School of Medicine, New Haven, CT, USA; ^2^Department of Psychology, Arizona State University, Tempe, AZ, USA; ^3^VA Connecticut Healthcare System, West Haven, CT, USA; ^4^Connecticut Council on Problem Gambling, Guilford, CT, USA; ^5^Problem Gambling Services of Connecticut, Middletown, CT, USA; ^6^Department of Neurobiology, Yale School of Medicine, New Haven, CT, USA; ^7^Child Study Center, Yale School of Medicine, New Haven, CT, USA

**Keywords:** alcohol, cigarette, impulsivity, marijuana, sensation seeking, smoking

## Abstract

*Background and aims:* Perceived parental permissiveness toward gambling may relate to adolescents’ engagement in various risky behaviors. To examine this possibility, we analyzed data from a high-school based risk-behavior survey to assess relationships between perceived parental permissiveness toward gambling and adolescent gambling behavior, substance use and related problems. We also evaluated predictions that relationships between perceived parental permissiveness toward gambling and risky behaviors would be particularly strong amongst adolescents reporting high sensation-seeking or impulsivity. *Methods:* High-school students (*n* = 2,805) provided data on risky behaviors, perceived parental permissiveness toward gambling, impulsivity and sensation-seeking. Bivariate and logistic regression analyses were conducted to examine relationships with gambling and alcohol, cigarette and marijuana use. *Results:* Perceived parental permissiveness toward gambling related significantly to adolescent gambling, all substance-use behaviors as well as alcohol and drug problems. There were significant parental-permissiveness-by-sensation-seeking interactions in multiple models. Relationships between perceived parental permissiveness toward gambling and alcohol-use frequency were particularly strong among those with high sensation-seeking. This relationship also applied to gambling and heavy cigarette smoking, albeit to a lesser extent. Impulsivity related strongly to drug problems among those who perceived their parents to be more and less permissive toward gambling. *Discussion and conclusions:* These findings support the relevance of perceived parental permissiveness toward gambling to adolescent risky behaviors. Parenting perceived as less permissive toward gambling appeared to have protective effects on gambling, alcohol and cigarette use, even among those with high sensation-seeking. Reducing parental permissiveness toward gambling may be a valuable intervention goal, particularly for parents of sensation-seeking adolescents.

## INTRODUCTION

Adolescent gambling is prevalent with estimates of past-year gambling ranging from 50–90% (Derevensky, Gupta & Winters, 2003; Welte, Barnes, Tidwell & Hoffman, 2008). Adolescents are 2–4 times more likely than adults to experience gambling problems (Derevensky et al., 2003). Nomenclature differences across studies on adolescent gambling may partly explain wide ranges in prevalence findings (Derevensky et al., 2003). In the present study, gambling was defined as any game you bet on for money or anything else of value, which encompasses a variety of activities including sports betting, casino gambling and lotteries.

Gambling has been associated with use of addictive substances, which may partly explain adverse outcomes associated with adolescent gambling. Adolescents who gamble are more likely to use alcohol (Duhig, Maciejewski, Desai, Krishnan-Sarin & Potenza, 2007), cigarettes and marijuana (Barnes, Welte, Hoffman & Tidwell, 2009). Further, frequent gambling and gambling-related problems among adolescents are related to heavy use of alcohol, cigarettes and marijuana as well as substance-related problems (Barnes et al., 2009). Identifying factors influencing adolescent gambling may help with prevention strategies aimed at reducing a wide range of risky behaviors during adolescence.

Multiple factors may influence adolescents’ decisions to engage in risky behaviors. Social learning theory suggests that attitudes predict behaviors and that attitudes are influenced by important individuals such as parents (Akers & Lee, 1996). Conceivably, parental attitudes may influence an offspring’s initial engagement in gambling as well as other risky behaviors (Akers & Lee, 1996). Thus, permissive parental attitudes are likely to increase risk of adolescent engagement in risky behaviors whereas less permissive attitudes should exert a protective effect, decreasing likelihood of adolescent risky behaviors. Accordingly, for cigarette smoking, protective effects of perceived parental disapproval have a stronger influence on adolescent decisions regarding smoking than peer and parental smoking (Kong et al., 2012; Sargent & Dalton, 2001). In contrast, perceived parental permissiveness relates to heavier cigarette smoking (Sargent & Dalton, 2001) and alcohol use (Fairlie, Wood & Laird, 2012). In a recent study, children’s ratings of their parents’ rearing practices predicted their drinking behavior prospectively to a greater extent than parents’ own ratings, supporting the validity of offspring ratings of parenting variables (Latendresse et al., 2009).

Although studies have examined perceived parental permissiveness towards substance-use behaviors, none to date have examined how perceived parental attitudes toward gambling influence adolescent engagement in risky behaviors. Since gambling and substance use are closely related (Leeman & Potenza, 2012), it is possible that perceived parental permissiveness toward gambling will relate both to gambling and substance use behaviors. Alternatively, perceived parental permissiveness toward gambling may relate more specifically to gambling behaviors. To our knowledge, these possibilities have not been examined directly. Considering the importance of parental attitudes, it would be valuable to assess the extent to which perceived parental attitudes toward gambling are specific or apply to multiple risky behaviors.

Parental controls may be of particular importance for offspring with higher levels of sensation seeking and impulsivity. Sensation-seeking has been defined as the pursuit of “varied, novel, complex, and *intense* sensations and experiences, and the willingness to take physical, social, *legal,* and *financial* risks for the sake of such experiences” (Zuckerman, 1994, p. 27, emphasis in original). Impulsivity has been defined as “a predisposition toward rapid, unplanned reactions to internal or external stimuli with diminished regard to the negative consequences of these reactions to the impulsive individual or others” (Moeller, Barratt, Dougherty, Schmitz & Swann, 2001; Brewer & Potenza, 2008). Sensation-seeking and impulsivity are related but distinct constructs (Ersche, Turton, Pradhan, Bullmore & Robbins, 2010) with disparate neurodevelopmental features and differing developmental trajectories (Steinberg et al., 2008). Sensation-seeking and impulsivity are particularly common among adolescents, partly because neural circuitry facilitating higher-order self-regulation has not fully developed (Potenza, 2013).

Impulsivity and sensation-seeking have both been associated with risky behaviors during adolescence (King, Fleming, Monahan & Catalano, 2011). Specifically, adolescent sensation-seeking and impulsivity are associated with substance use cross-sectionally (Chassin, Flora & King, 2004; Martin et al., 2002) and predict substance use problems longitudinally (Chassin et al., 2004; Wanner, Vitaro, Ladouceur, Brendgen & Tremblay, 2006). Impulsivity is related to gambling initiation (Auger, Lo, Cantinotti & O’Loughlin, 2010) and subsequent problem gambling (Vitaro, Arseneault & Tremblay, 1999). Adolescent sensation-seeking is related to problem gambling severity cross-sectionally (Gupta, Derevensky & Ellenbogen, 2006). Relationships between risky behaviors and impulsivity/sensation-seeking among young people may be reciprocal (Quinn, Stappenbeck & Fromme, 2011). Impulsivity and sensation-seeking measured in late adolescence predicted heavy drinking during undergraduate years and heavy drinking then predicted subsequent increases in sensation-seeking and impulsivity among undergraduates (Quinn et al., 2011).

Perceived parental permissiveness is likely problematic overall, but particularly among impulsive (Patock-Peckham & Morgan-Lopez, 2006) and/or sensation-seeking youth. Permissive parenting has been associated with sensation-seeking among elementary-school children (Xu, Farver & Zhang, 2009) and sensation-seeking, rebelliousness and substance use among adolescents (Hayes, Hudson & Matthews, 2004).

In separate statistical models along with other potential correlates, we analyzed relationships between perceived parental permissiveness toward gambling and adolescents’ reports of their gambling and substance use, along with gambling- and substance-related problems. These statistical models were expected to provide valuable information regarding the extent to which gambling, substance use and related problems are associated with similar or different correlates. We also predicted interactions, such that relationships between perceived parental permissiveness toward gambling and risky behaviors would be strongest among adolescents reporting high sensation-seeking or impulsivity.

The present study is unique in multiple respects. Few studies have addressed perceived parental permissiveness in relation to multiple risky behaviors, and we are aware of no such studies that examined perceived parental permissiveness towards gambling. This approach of relating a probable correlate of gambling to other types of risky behaviors is novel. Studies examining impulsivity and sensation-seeking as possible moderators of relationships between parental permissiveness and risky behaviors are rare. The present study is an extension of Leeman, Hoff, Krishnan-Sarin, Patock-Peckham and Potenza (in press), who found that both sensation-seeking and impulsivity related significantly to substance use and impulsivity related to gambling. Impulsivity had stronger relationships to gambling and drug-related problems than did sensation-seeking. The present study extends Leeman et al. (in press) by examining relationships between perceived parental permissiveness toward gambling and risky behaviors, along with possible moderation of these effects by impulsivity or sensation-seeking.

## METHODS

### Sample

Recruitment for the parent study has been described in prior reports (e.g., Schepis et al., 2008). All public four-year non-special education and non-vocational secondary schools in the state of Connecticut were invited to participate. The schools were offered a risk behavior summary assessment of their students. Study staff contacted schools expressing interest in the study. A targeted selection was undertaken for geographic regions not represented sufficiently. The final sample for the parent study included schools from each geographic quadrant and three clusters of district reference groups (DRGs), which are based on the socio-economic status of households in these districts. The final parent study sample (*n* = 4523) was similar to key Connecticut demographics for this age group. The sample for the present study (*n* = 2805) (Table 1) was comprised of participants giving complete responses for all variables included in the statistical models (i.e., perceived parental permissiveness toward gambling, sensation-seeking, impulsivity and demographics) and at least one risky behavior variable (i.e., gambling and substance use variables).

**Table 1.** Sample characteristics

Variable	Percent/Mean *(SD)*
Percent female	55.8%
Mean age (SD)	15.84 years (1.23)
Race	78% White
	5.3% African-American
	3% Asian
	4.3% multiple races
	9.3% other
Ethnicity	88.5% non-Hispanic/Latin
	11.5% Hispanic/Latin
Living situation	74.1% with two parents
	21.7% with one parent
	4.2% other
Lifetime alcohol users (at least 1 drink)	68.8%
Having at least 1 binge-drinking day in past 30 days	30.6%
Alcohol consumption frequency in past 30 days	52.6% none
	30.5% one–five days (infrequent)
	16.9% six days or more (frequent)
Reporting a lifetime problem with alcohol	3.9%
Lifetime cigarette smokers	37.1%
Cigarette use frequency in past 30 days	80.8% none
	12.8% up to seven per day (infrequent)
	6.4% eight or more per day (frequent)
Lifetime marijuana users	38%
Smoking marijuana at least once in past 30 days	22%
Reporting a lifetime problem with drug use	4.6%
Engaging in any gambling in past 12 months	91.5%
Hours spent gambling in an average week	88.3% an hour or less (infrequent)
	11.7% two hours or more (frequent)
Endorsing one or more criteria for pathological gambling in past 12 months	13.7%
Mean impulsivity score *(SD)*	3.41 (2.20)
Mean sensation-seeking score *(SD)*	6.59 (2.91)
Mean score of perceived parental permissiveness toward gambling (1–5 scale, higher score = more permissive)	2.35 (0.92)

### Procedure

Researchers distributed the surveys during school assemblies or specific classes (e.g., health or English). Students were given instructions to avoid including information that might identify them. Participating students were given a pen for completing the survey. Rate of refusal was less than 1%.

### Measures

As in prior investigations from the parent study (e.g., Leeman et al., in press; Schepis et al., 2008), categorical versions of risky behavior variables (i.e., gambling, alcohol, cigarette and marijuana use, along with related problems) were created for analytic purposes due to limited variability of responses to these items. Use of similar groupings also allows for continuity between the present study and other manuscripts published from the parent study data.

*Gambling.* For gambling items, students were given the following definition of gambling: “any game you bet on for money OR anything else of value”. Students were asked to report their number of hours spent gambling or placing bets in an average week. Those reporting gambling ≤1 hour per week or who reported no past 12-month involvement in any type of gambling were classified as infrequent gamblers, whereas those reporting gambling ≥2 hours per week were classified as frequent gamblers.

*Perceived parental permissiveness regarding gambling.* Students responded to the following item: “How do you think your parents would feel about you gambling, even once or twice, over the next 12 months?” Five response options were given, ranging from “strongly disapprove” (scored “1”) to “strongly approve” (scored “5”).

*Alcohol.* We created two variables capturing alcohol use. Students first indicated whether they have ever had a drink of alcohol in their lifetime besides a few sips. Lifetime drinkers reported past-30-day frequency of any alcohol consumption, responses to which were converted into a 3-level variable: no use, infrequent (1–5 days) and frequent use (≥6 days). Students reported their past-30-day frequency of binge-drinking (i.e., males: 5 or more drinks in a row, females: 4 or more) as well. We created a binary (any/none) variable concerning past-30-day binge-drinking.

*Marijuana and cigarettes.* Students indicated whether they have ever smoked marijuana. Those who have smoked reported their frequency of use in the prior 30 days. We combined these items into a single binary variable capturing past-30-day any/no use. Similarly, students first reported whether they had ever smoked cigarettes. Those who had smoked reported their average cigarette use per day over the prior 30 days. These items were collapsed into one variable with students grouped as non-smokers, light (≤7 per day) and heavy smokers (≥8 per day).

*Gambling and substance-related problems.* Students reported in separate items whether they ever had a problem with alcohol or drugs: “Do you now have, or have you ever had, an alcohol problem?” “Do you now have, or have you ever had, any kind of a drug problem?” Questions regarding *DSM-IV-TR* pathological-gambling criteria were assessed with items from the Massachusetts Gambling Screen (Shaffer, LaBrie, Scanlan & Cummings, 1994). Those reporting past-year gambling without endorsing any DSM-IV criteria were classified as low-risk gamblers, where individuals meeting 1 or more DSM-IV criteria were considered at-risk/problem gamblers (e.g., Kundu et al., 2013; Leeman et al., in press).

*Sensation-seeking and impulsivity.* Items from the Zuckerman–Kuhlman Personality Questionnaire (Zuckerman, Kuhlman, Joireman, Teta & Kraft, 1993) were used to assess these constructs. The measure included 19 true/false questions pertaining to characteristics associated with sensation-seeking (11 items, a = .78) or impulsivity (8 items, a = .70). Responses indicative of sensation-seeking or impulsivity were scored “1” with opposing responses scored “0”.

*Demographics*. Students reported their age, race/ethnicity, gender and current living situation (i.e., living with no parents, with 1 or 2 parents). The living situation variable was included in statistical models to ensure that any effect of perceived parental permissiveness toward gambling was not attributable to absence of 1 or more parents. For logistic regressions, two dummy variables were created comparing living with 1 and no parents to other living situations, making living with two parents the reference category.

### Data analysis

IBM SPSS, version 19 was used to conduct all analyses. Prior to the main analyses, we examined item distributions; bivariate correlations among variables; chi-square analyses and *t*-tests to compare variables between students included in the present study and those omitted due to missing data.

Logistic regression models were tested for binary variables and multinomial logistic regression models for the 3-level categorical variables. Students having complete data were included in models for gambling frequency and substance use (i.e., overall frequency of alcohol use, frequency of binge-drinking, marijuana and cigarette use). Abstainers over the past year for gambling and on a lifetime basis for substances) were excluded from models for related problems (i.e., gambling, alcohol and drugs). Entry of variables into models was in a two-step process: individual variables in an initial step and interaction terms in a second step. We tested two-way interactions of perceived parental permissiveness toward gambling with sensation-seeking and with impulsivity. Sensation-seeking and impulsivity scores were mean-centered to reduce potential collinearity with the interaction terms (Aiken & West, 1991). Interactions having *p* values ≥.05 were dropped from models and the models were then re-tested. Therefore, final models for gambling frequency and substance use included perceived parental permissiveness toward gambling, sensation-seeking, impulsivity, demographics and remaining interactions. A similar approach was taken for related problems with the exception that a variable capturing gambling frequency or substance use was also included. Model goodness of fit was determined with chi-square comparisons of –2 log likelihoods between constant-only versus final models, along with Hosmer and Lemishow tests for logistic regressions and deviance statistics, plus Pearson tests for multinomial logistic regressions. Plots of simple regression equations at individual variable values 1 *SD* above and below 0 were used to interpret significant interactions (Aiken & West, 1991). Due to testing multiple models with multiple comparisons, we adopted an alpha level of *p* ≤ .01 for significance for all models.

### Ethics

Introductory letters were mailed to parents. Those who preferred that their child not participate were requested to contact the school. In absence of contact, parental consent was assumed. Students were told that participation was voluntary. Students who preferred not to participate and those who did not have parental consent either sat quietly or completed alternate work. Yale’s Institutional Review Board and participating schools approved consent procedures. The study was conducted in accordance with the Declaration of Helsinki and the Health Insurance Portability and Accountability Act (HIPAA).

## RESULTS

### Preliminary analyses

Using all available data, we compared those included in the studied sample with those not included due to missing data. Excluded individuals were more likely to be male, non-white and of Hispanic/Latino ethnicity; reside with one or no parents; report frequent cigarette smoking, and marijuana use; report lifetime histories of alcohol- and drug-use problems and score higher on impulsivity. The highest correlation was between impulsivity and sensation-seeking (*r* = .58, *p* < .001). Thus, no correlations were high enough to raise concern about collinearity. Tests indicated good fit to the data for all regression models.

### Regression models for gambling and substance use

**Figure 1.**
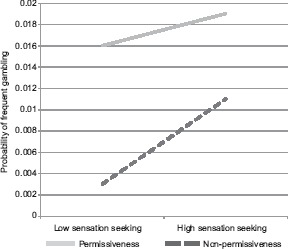
Sensation seeking × parental permissiveness for gambling *Note:* Results of simple regression equations at individual variable values 1 *SD* above and below 0.

*Gambling.* In a logistic regression, there were significant effects of perceived parental permissiveness toward gambling, sensation-seeking and impulsivity (Table 2). Significant effects of sensation-seeking and perceived parental permissiveness toward gambling should be interpreted in light of a significant interaction. Perceived parental permissiveness related to gambling frequency at all levels of sensation-seeking; however, as predicted, relationships were somewhat stronger among those with high sensation-seeking. In addition to this predicted effect, among those who perceived their parents as being non-permissive regarding gambling, sensation-seeking had a strong relationship to gambling frequency (Figure 1). Gender (males higher) and the dummy variable comparing living with no parents versus other living situations were also significant.

**Figure 2.**
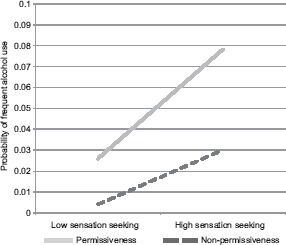
Sensation seeking × parental permissiveness for alcohol *Note:* Results of simple regression equations at individual variable values 1 *SD* above and below 0.

*Alcohol.* According to logistic regression (Table 2) and multinomial logistic regression (Table 3) results, perceived parental permissiveness toward gambling, impulsivity and sensation-seeking were significantly and positively associated with binge-drinking and more frequent alcohol consumption overall. The impulsivity effect for overall frequency pertained only to the comparison between frequent consumption and no use. Effects of sensation-seeking and perceived parental permissiveness toward gambling in the overall frequency model should be interpreted in light of a significant interaction, which pertained only to the comparison between frequent and no alcohol use. As predicted, the relationship between perceived parental permissiveness toward gambling and frequent alcohol use was considerably stronger among those with high sensation-seeking compared to those with low sensation-seeking. This effect was more dramatic than it was for gambling. In addition to the predicted effect, among those who considered their parents to be less permissive toward gambling, high sensation-seeking was associated with a higher probability of frequent alcohol use. Likelihood of frequent alcohol use was about the same for these students as it was for those with low sensation-seeking who perceived their parents to be permissive about gambling (Figure 2). Older and white students were more likely to have endorsed binge-drinking at least once in the past 30 days and endorsed more frequent consumption overall. Being Hispanic/Latino was significantly associated with binge-drinking and marginally associated with overall alcohol-use frequency. Gender was related to overall frequency due to an association between female gender and infrequent compared to no drinking.

**Table 2.** Logistic regression models for binge-drinking of alcohol, marijuana use and gambling

Variable	Binge-drinking of alcohol			Marijuana use			Gambling		
	O.R.	95% C.I.for O.R	*p*-value	O.R.	95% C.I.for O.R	*p*-value	O.R.	95% C.I.for O.R	*p*-value
Age	1.47	1.36–1.59	<.001	1.31	1.20–1.42	<.001	1.18	1.03–1.35	.020
Gender	0.84	0.70–1.01	.070	1.03	0.89–1.26	.751	5.41	3.42–8.52	<.001
Race	1.93	1.45–2.57	<.001	1.60	1.18–2.17	.003	0.95	0.60–1.57	.907
Ethnicity	2.03	1.45–2.84	<.001	1.35	0.93–1.90	.120	1.59	0.91–2.76	.101
Living with one parent versus other	0.91	0.73–1.13	.485	1.22	0.98–1.57	.073	1.61	1.06–2.38	.024
Living with no parents versus other	1.47	0.95–2.26	.084	2.39	1.49–3.58	<.001	4.30	2.32–7.97	<.001
Impulsivity	1.11	1.06–1.17	<.001	1.12	1.06–1.19	<.001	1.15	1.04–1.27	.007
Sensation-seeking	1.19	1.14–1.24	<.001	1.18	1.13–1.24	<.001	1.13	1.03–1.23	.009
Perceived parental permissiveness	1.68	1.51–1.87	<.001	1.50	1.34–1.68	<.001	1.76	1.45–2.14	<.001
Sensation-seeking-by-parental-permissiveness interaction		ns			ns		0.91	0.86–0.97	.002

*Note:* Gender: male coded 1, female coded 0; race: white coded 1, non-white coded 0; ethnicity: Hispanic/Latino coded 1, non-Hispanic/Latino coded 0; O.R. = odds ratio; C.I. = 95% confidence interval.

**Table 3.** Multinomial logistic regression models for overall frequency of alcohol use and heaviness of cigarette use

Variable	Frequent vs. no alcohol consumption			Infrequent vs. no alcohol consumption			Heavy cigarette use vs. no cigarette use			Light cigarette use vs. no cigarette use		
	O.R.	95% C.I. for O.R	*p*-value	O.R.	95% C.I. for O.R	*p*-value	O.R.	95% C.I. for O.R	*p*-value	O.R.	95% C.I. for O.R	*p*-value
Age	1.66	1.50–1.83	<.001	1.32	1.22–1.42	<.001	1.42	1.23–1.63	<.001	1.33	1.21–1.47	<.001
Gender	0.75	0.59–0.95	.017	0.72	0.60–0.87	.001	0.88	0.62–1.23	.451	0.56	0.44–0.73	<.001
Race	1.70	1.19–2.43	.004	1.56	1.19–2.05	.001	1.62	0.97–2.71	.065	1.28	0.89–1.84	.182
Ethnicity	1.56	1.02–2.40	.043	1.61	1.15–2.26	.006	1.91	1.11–3.28	.020	0.88	0.56–1.38	.571
Living with 1 parent vs. other	1.14	0.86–1.50	.371	1.01	0.81–1.27	.911	1.71	1.18–2.45	.005	1.21	0.92–1.60	.180
Living with no parents vs. other	1.54	0.91–2.60	.109	0.81	0.49–1.33	.402	3.18	1.75–5.79	<.001	1.25	0.70–2.23	.445
Impulsivity	1.14	1.07–1.22	<.001	1.02	0.97–1.08	.396	1.19	1.08–1.31	<.001	1.12	1.05–1.19	.001
Sensation-seeking	1.30	1.23–1.38	<.001	1.20	1.15–1.25	<.001	1.29	1.17–1.43	<.001	1.24	1.17–1.31	<.001
Perceived parental permiss.	2.13	1.84–2.46	<.001	1.48	1.34–1.65	<.001	2.69	2.18–3.32	<.001	1.33	1.14–1.56	<.001
Sensation-seeking–by-parental-permissiveness interaction	0.93	0.89–0.98	.002	0.98	0.95–1.02	.347	0.88	0.83–0.94	<.001	0.98	0.93–1.03	.373

*Note:* Gender: male coded 1, female coded 0; race: white coded 1, non-white coded 0; ethnicity: Hispanic/Latino coded 1, non-Hispanic/Latino coded 0; O.R. = odds ratio; C.I. = 95% confidence interval.

*Marijuana.* In a logistic regression, there were significant effects of perceived parental permissiveness toward gambling, impulsivity and sensation-seeking (Table 2). Age, race (white students more likely to smoke) and living with no parents versus other living situations were also significant. There were no significant interactions.

**Figure 3.**
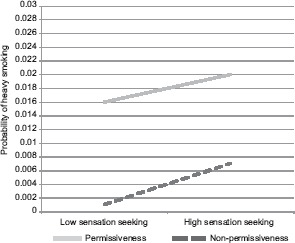
Sensation seeking × parental permissiveness for cigarettes *Note:* Results of simple regression equations at individual variable values 1 *SD* above and below 0.

*Cigarettes*. In a multinomial regression, there were significant effects of perceived parental permissiveness toward gambling, impulsivity and sensation-seeking (Table 3). Associations involving perceived parental permissiveness and sensation-seeking should be interpreted in light of a significant interaction. Similar to the same interaction in the gambling model, perceived parental permissiveness toward gambling was associated with heavy smoking at all levels of sensation-seeking. However, as predicted, relationships were somewhat stronger among those with high sensation-seeking. In addition to this predicted effect, among those who perceived their parents as being non-permissive toward gambling, sensation-seeking was associated with a greater probability of heavy smoking (Figure 3). Age, gender and living with fewer than two parents also related significantly to cigarette use (Table 3). The gender effect was predominantly due to a relationship between female gender and light as compared to no smoking.

### Regression models for gambling and substance-related problems

Current abstainers were excluded from models for gambling- and substance-related problems. In a model of gambling-related problems, perceived parental permissiveness toward gambling was not significant, whereas impulsivity was. Gender (males higher) was the only significant demographic variable, and gambling frequency was significant (Table 4). There were no significant interactions.

In a model for lifetime alcohol problems, there were significant effects of perceived parental permissiveness toward gambling and impulsivity, which should be interpreted in light of a near significant interaction. Perceived parental permissiveness toward gambling had somewhat stronger relationships to probability of a lifetime alcohol problem among more impulsive students. In addition to this predicted relationship, among those who perceived their parents to be less permissive toward gambling, high impulsivity strongly related to probability of a lifetime alcohol problem. Probability of an alcohol problem among these students was similar to that of low impulsivity students who perceived their parents to be more permissive regarding gambling. The variable comparing living with no parents to other situations and frequency of alcohol consumption also had significant relationships to alcohol problem history (Table 4).

**Table 4.** Logistic regression models for alcohol, drug and gambling-related problems

Variable	Alcohol			Drug use			Gambling		
	O.R.	95% C.I. for O.R.	*p*-value	O.R.	95% C.I. for O.R.	*p*-value	O.R.	95% C.I. for O.R.	*p*-value
Age	1.13	0.95–1.34	.167	1.09	0.89–1.32	.405	0.99	0.88–1.11	.856
Gender	1.06	0.70–1.62	.773	1.18	0.75–1.88	.475	3.08	2.23–4.26	<.001
Race	1.36	0.70–2.67	.365	1.05	0.52–2.13	.885	0.94	0.63–1.40	.755
Ethnicity	1.39	0.68–2.82	.367	1.34	0.63–2.87	.450	1.43	0.89–2.30	.144
Living situation: with one parent vs. other	0.96	0.61–1.68	.959	1.38	0.82–2.31	.225	0.86	0.61–1.23	.401
Living situation: with no parents vs. other	3.83	1.99–7.38	<.001	2.56	1.17–5.59	.018	1.34	0.73–2.47	.344
Frequency of engagement	2.37	1.36–4.12	.002	4.39	2.33–8.30	<.001	4.98	3.37–7.37	<.001
Impulsivity	1.20	1.06–1.37	.003	1.28	1.11–1.47	.001	1.13	1.04–1.23	.003
Sensation-seeking	1.03	0.94–1.13	.533	1.05	0.94–1.16	.372	1.04	0.98–1.11	.194
Perceived parental permissiveness	1.53	1.17–2.01	.002	1.47	1.09–1.97	.007	0.99	0.85–1.16	.918
Impulsivity-by-permissiveness interaction	0.86	0.77–0.97	.012	0.81	0.71–0.92	<.001		ns	

*Note:* Gender: male coded 1, female coded 0; race: white coded 1, non-white coded 0; ethnicity: Hispanic/Latino coded 1, non-Hispanic/Latino coded 0; O.R. = odds ratio; C.I. = 95% confidence interval; frequency of engagement is frequency of alcohol use for alcohol problems, frequency of marijuana use for drug problems and frequency of gambling for gambling problems; for frequency of alcohol use, the comparison between frequent and no use is reported here, the comparison between infrequent and no use was not significant. ns = non-significant.

**Figure 4.**
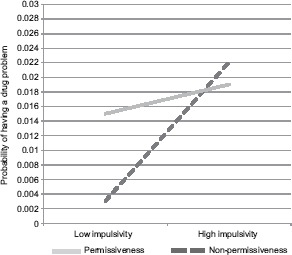
Impulsivity × parental permissiveness for drug problems *Note:* Results of simple regression equations at individual variable values 1 *SD* above and below 0.

In a model of lifetime drug problems, perceived parental permissiveness toward gambling and impulsivity were significant; however, these effects should be interpreted in light of a significant interaction (Table 4). As predicted, the relationship between perceived parental permissiveness toward gambling and lifetime probability of a drug problem was somewhat stronger among more impulsive students. However, in addition to the predicted effect, among those who perceived their parents to be less permissive about gambling, impulsivity had a strong relationship to lifetime probability of a drug problem. In fact, lifetime probability of a drug problem was roughly equivalent among impulsive students who perceived their parents to be permissive and non-permissive about gambling (Figure 4). Past-30-day marijuana use was also significantly associated with drug problem history; however, there were no significant demographic variables.

## DISCUSSION AND CONCLUSIONS

Based on social-learning theory (Akers & Lee, 1996; Bandura & Walters, 1963), we predicted strong relationships between perceived parental permissiveness toward gambling and adolescent risky behaviors (i.e., gambling, substance use and related problems). This prediction was confirmed for all variables except gambling-related problems. We predicted further that relationships between perceived parental permissiveness toward gambling and risky behaviors would be strongest among impulsive or sensation-seeking students. This prediction was partly confirmed. Relationships between perceived parental permissiveness toward gambling and multiple risky behaviors (i.e., gambling, alcohol use and cigarette smoking) were stronger among students who reported higher sensation-seeking. Surprisingly, there were no significant interactions with respect to gambling-related problems. There were also no significant interactions involving impulsivity for gambling or substance use. However, there was a parallel near-significant finding in which the relationship between perceived parental permissiveness regarding gambling and lifetime history of an alcohol-use problem was somewhat stronger among more impulsive individuals.

In addition to these predicted findings, sensation-seeking had strong relationships to gambling, alcohol use and cigarette smoking among those who perceived their parents as being less permissive toward gambling. Similarly, in a near significant interaction, impulsivity related strongly to alcohol problem history among those who viewed their parents as being less permissive toward gambling. Contrary to predictions, impulsivity was associated with drug use problems among those who perceived their parents to be more and less permissive toward gambling.

Among those with high sensation-seeking, engagement in gambling, alcohol use and cigarette smoking was lower among those who perceived their parents as being less permissive toward gambling, compared to those who viewed their parents as being permissive toward gambling. Thus, parenting perceived as being less permissive appeared to have a protective effect even among higher-risk students with sensation-seeking tendencies; however, this protective effect did not apply to impulsive adolescents with regard to drug use problems. These findings follow results from a personality-targeted intervention (Conrod, Castellanos & Mackie, 2008), which was found to be particularly effective in preventing the negative impact of sensation-seeking on adolescent binge-drinking. Thus, the impact of sensation-seeking on substance-use behavior may be more malleable based on environmental factors than the impact of impulsivity is.

These findings extend prior results supporting relationships between perceived parental permissiveness and risky behaviors and potential protective effects of less permissive parenting (Fairlie et al., 2012; Sargent & Dalton, 2001), even among higher-risk adolescents (Pieters et al., 2012; Xu et al., 2009). Parental external control (van der Vorst, Engels, Meeus & Dekovic, 2006) may have particularly high protective value for youth at risk for self-control issues (Pieters et al., 2012), such as those with high sensation-seeking.

Interestingly, perceived parental permissiveness toward gambling related strongly not only to gambling, but also to substance-use behavior and related problems. These findings further support observations of strong relationships between gambling and substance use (Leeman & Potenza, 2012). Our findings further suggest that parental attitudes toward gambling may act as a proxy for parental attitudes toward other addictive behaviors and perhaps to other types of risky behaviors. Speculatively, permissive parental attitudes toward gambling may contribute to the inordinately high prevalence of this behavior among adolescents demonstrated in this as well as in prior studies (Derevensky et al., 2003; Welte et al., 2008).

This study has several strengths, including a large sample size. Inclusion of multiple gambling and substance-use variables in the same study is relatively unique and allows for assessment of consistency with respect to correlates of these behaviors. Inclusion of sensation-seeking and impulsivity in the same statistical models permitted observation of similarities and differences regarding associations with risky behaviors and interactions with perceived parental permissiveness toward gambling.

The study also had limitations. The goal of the parent study was breadth (arguably more so than depth). Accordingly, multiple health behaviors and possible correlates were assessed. Several constructs were examined using single items, such as perceived parental permissiveness toward gambling and problems related to lifetime drug and alcohol problems. That we identified significant relationships involving perceived parental permissiveness despite the single-item measure and focus on gambling suggests the potential robustness of this construct as a correlate of risky behaviors. There was a considerable amount of missing data. In several respects, participants with missing data appeared to be at greater risk (e.g., gambled, smoked cigarettes and marijuana more often) than those having complete data. Nonetheless, we found multiple significant relationships between varied risk factors and gambling/substance use. Complete data from these students could have further strengthened results of this study.

The present findings highlight the importance of parental influence on gambling and substance use. Specifically, our findings show potentially damaging effects of parental permissiveness toward gambling, as well as the protective value of less permissive parenting, even among higher-risk youth. Consequently, simultaneous intervention with parents to reduce permissiveness appears to be a valuable intervention goal (Fairlie et al., 2012). Accordingly, parent-based interventions have already shown efficacy in reducing alcohol consumption among late adolescents (i.e., first-year college students) (Turrisi et al., 2013).
